# Editorial: Immunotherapy in urologic malignancies

**DOI:** 10.3389/fruro.2025.1582134

**Published:** 2025-03-26

**Authors:** Jayalekshmi Jayakumar, Arya Mariam Roy

**Affiliations:** ^1^ Department of Internal Medicine, The Brooklyn Hospital Centre, Brooklyn, NY, United States; ^2^ Department of Oncology, The Ohio State University Comprehensive Cancer Centre, Columbus, OH, United States

**Keywords:** urology, bladder cancer, kidney cancer, prostate cancer, immunotherapy

Urologic malignancies, including prostate, kidney, bladder, and testicular cancers, have historically presented significant therapeutic challenges, with conventional modalities such as chemotherapy and radiation often providing limited clinical benefit (1, 2). Immunotherapy has emerged as a transformative approach, harnessing the immune system’s intrinsic ability to recognize and eliminate malignant cells through various mechanisms. Among these, immune checkpoint inhibitors have demonstrated notable clinical efficacy by targeting regulatory proteins that suppress immune activation and impede tumor antigen recognition. By overcoming these immunosuppressive barriers, checkpoint inhibitors enhance anti-tumor immune responses, leading to durable clinical outcomes in a subset of patients (3). As a result, immunotherapy has assumed a central role in the management of urologic cancers, contributing to improved survival rates and quality of life. This Research Topic highlights studies examining the immune microenvironment of urologic malignancies, which may inform the development of novel immunotherapeutic strategies and identify predictors of response to specific immunotherapy agents in these cancers.

A major challenge in immunotherapy is the absence of standardized algorithms or predictive tools to assess responses to novel immunotherapeutic agents. Pezeshki et al. introduced the “microcancer” concept, utilizing 3D tumor spheroid cultures to predict responses to immune checkpoint inhibitor therapy in renal and bladder cancers. Unlike traditional models that rely on established tumor cell lines, this ‘immunotumoroid model’ was developed using patient-derived tumor cells obtained from surgical or biopsy specimens. Following tissue dissociation, the model incorporated tumor cells, stromal cells, and tumor-infiltrating lymphocytes, effectively capturing the *in vivo* heterogeneity of the tumor microenvironment, including immune cells and the extracellular matrix. This platform also allowed for scalable testing with standardized measurements. Although initial results from a small cohort were promising, the model’s external validity remains limited. The authors suggest that future studies incorporating combination therapies may enhance the model’s predictive capability and potentially address a key limitation of immune checkpoint inhibitors—therapeutic resistance.

In contrast to the immuno-tumoroid model, Betancor et al. developed a gene expression score (GES) to predict clinical benefit from the anti-PD-1 antibody nivolumab in patients with advanced renal cell carcinoma. This model utilized pooled data from the CheckMate-009, CheckMate-010, and CheckMate-025 clinical trials. The GES was based on the expression levels of three genes: HMGA1, NUP62, and ARHGAP42. The authors not only validated the score but also assessed its predictive value in the CheckMate-025 trial, where it demonstrated significant interactions with treatment outcomes. Further analysis explored the molecular, clinical, and immune characteristics associated with favorable and unfavorable GES results, linking these scores to specific tumor subtypes, patterns of immune cell infiltration, and the extent of fibrosis. These findings offer insights that could inform future therapeutic strategies, including combination therapies targeting both immune checkpoints and cancer-associated fibroblasts. This model integrates both prognostic and predictive capabilities, providing a standardized approach to identifying patients who are most likely to benefit from nivolumab therapy. If validated through larger prospective studies, the GES could serve as a valuable tool for optimizing immunotherapy strategies in renal cell carcinoma, offering a more personalized and effective treatment approach than existing biomarkers.

While these studies propose potential predictive tools for immune checkpoint inhibitor therapies, a case report by Zhang et al. introduced a novel treatment approach for epithelioid hemangioendothelioma of the prostate with lung and lymph node metastases. The authors described alternating nivolumab therapy with ipilimumab and liposomal doxorubicin, resulting in a significant partial response. Given the rarity of prostatic hemangioendothelioma and the absence of standardized treatment guidelines, this strategy of alternating immunotherapy with chemotherapy presents a promising therapeutic option that warrants further investigation in similar rare malignancies.

Radical cystectomy combined with pelvic lymph node dissection (PLND) remains the standard treatment for patients with non-metastatic bladder cancer at high risk of disease progression. PA-MSHA, a genetically modified and inactivated form of Pseudomonas aeruginosa expressing mannose-sensitive hemagglutinin, has demonstrated anti-tumor properties and has been used in several malignancies (4, 5). Zhang et al. evaluated the potential utility of PA-MSHA following PLND in radical cystectomy through a retrospective cohort study. Their findings suggest that PA-MSHA is safe and may improve overall survival, progression-free survival, and cancer-specific survival in patients undergoing radical cystectomy. However, the study’s small sample size and retrospective design limit its generalizability and raise concerns about potential bias. Larger prospective trials are necessary to validate these findings and establish the clinical relevance of PA-MSHA in this setting.

The studies presented in this Research Topic underscore the promising potential of immunotherapy in urologic cancers, paving the way toward more personalized and effective treatment strategies. Innovative models such as the immuno-tumoroid and GES represent significant advancements, though larger, well-designed studies are necessary to validate their clinical utility. The exploration of combination therapies for rare prostate cancer subtypes and the use of novel anti-tumor agents like PA-MSHA further highlight the expanding therapeutic landscape of immunotherapy in urologic malignancies. While these approaches show promise, they require rigorous validation through prospective clinical trials. If confirmed, these tools and strategies could revolutionize treatment paradigms, enhancing the accessibility and personalization of immunotherapy on a global scale. Ultimately, such advancements have the potential to improve survival outcomes and quality of life for patients with urologic cancers worldwide.
